# When parasites disagree: Evidence for parasite-induced sabotage of host manipulation

**DOI:** 10.1111/evo.12612

**Published:** 2015-03-10

**Authors:** Nina Hafer, Manfred Milinski

**Affiliations:** 1 Department of Evolutionary Ecology, Max-Planck-Institute for Evolutionary Biology, August-Thienemann-Strasse 2D-24306 Ploen, Germany

**Keywords:** Cestode, conflict, cooperation, copepod, experimental infections, parasite–parasite interactions

## Abstract

Host manipulation is a common parasite strategy to alter host behavior in a manner to enhance parasite fitness usually by increasing the parasite's transmission to the next host. In nature, hosts often harbor multiple parasites with agreeing or conflicting interests over host manipulation. Natural selection might drive such parasites to cooperation, compromise, or sabotage. Sabotage would occur if one parasite suppresses the manipulation of another. Experimental studies on the effect of multi-parasite interactions on host manipulation are scarce, clear experimental evidence for sabotage is elusive. We tested the effect of multiple infections on host manipulation using laboratory-bred copepods experimentally infected with the trophically transmitted tapeworm *Schistocephalus solidus*. This parasite is known to manipulate its host depending on its own developmental stage. Coinfecting parasites with the same aim enhance each other's manipulation but only after reaching infectivity. If the coinfecting parasites disagree over host manipulation, the infective parasite wins this conflict: the noninfective one has no effect. The winning (i.e., infective) parasite suppresses the manipulation of its noninfective competitor. This presents conclusive experimental evidence for both cooperation in and sabotage of host manipulation and hence a proof of principal that one parasite can alter and even neutralize manipulation by another.

Parasites can modify their host's phenotype to their own benefit. Such host manipulation is known from a wide range of both host and parasite taxa (Holmes and Bethel 1972; Poulin and Thomas 1999; Moore 2002, 2013; Poulin 2010), including humans (Flegr 2013). In parasites with complex life cycles, it usually enhances a parasite's chances to pass on to the next host at the appropriate time point (Parker et al. 2009). Before being able to infect the next host, some parasites lower their present host's predation susceptibility: premature predation even by the correct consecutive host would be fatal to the parasite (Koella et al. 2002; Hammerschmidt et al. 2009; Thomas et al. 2010; Dianne et al. 2011). Once the parasite is infective to the next host, manipulation increases transmission to that host, for example, by increasing the current host's predation susceptibility (Holmes and Bethel 1972; Poulin and Thomas 1999; Moore 2002, 2013; Poulin 2010). Such predation enhancement can also be a mere side effect of the parasite's draining energy from the host, forcing it to shift its trade-off between avoiding predation and decreasing hunger toward the latter (Milinski 1990).

Most experimental studies on host manipulation investigated the effect of a single infection on host behavior. In nature, hosts are usually infected by multiple parasites, typically from different species encountered sequentially (e.g., Kalbe et al. 2002). Manipulation by one parasite will affect every coinfecting parasite, even nonmanipulating ones (Milinski 2014). Do parasites react to manipulation of a coinfecting parasite? If interests coincide, the presence of a second manipulator might be beneficial. Potential costs could be shared or manipulation be enhanced increasing transmission probability. Correlational evidence suggests that multiple parasites may indeed strengthen each other's manipulation if they have the same aim (reviewed by Cézilly et al. 2014).

By contrast, two coinfecting parasites with incompatible aims have a conflict over host manipulation, either because they manipulate in different directions or one parasite manipulates whereas the other one does not manipulate if its interest is best served by the host's normal behavior. In both cases, one parasite would benefit from “sabotaging,” that is, partly or completely suppressing the manipulation by the other parasite (Thomas et al. 2002). Several studies provide correlational evidence for parasites being able to alter manipulation by another parasite. Most of these studies, however, used exclusively naturally infected hosts, making it impossible to decide whether the parasite really caused the observed alteration of host behavior (reviewed by Cézilly et al. 2014). To our knowledge, only two studies used experimental infections to obtain hosts with parasites that had different aims. Thomas et al. (2002) experimentally cured and reinfected trematode-infected gammarids, that is, small crustaceans, with nematodes that had appeared to sabotage manipulation by the trematodes in natural infections. The authors did, however, not find the previously observed sabotage. Dianne et al. (2010) experimentally infected gammarids with different stages of an acanthocephalan parasite and found suggestive evidence that the not yet infective stage might have sabotaged manipulation by the infective one. Both studies used wild-caught hosts, which might have encountered various other parasites before. One preliminary study infected laboratory-bred rats with two parasites known to affect the host's nervous system, including *Toxoplasma*, a common parasite also capable of manipulating human behavior. One parasite partly influenced the effect of manipulation by another; however, since no significant differences were found between the different parasites, it remains elusive to which extend there was actual conflict between the parasites or whether this observation was a mere side effect (De Queiroz et al. 2013). Thus, sabotage may exist but was not stringently shown under experimentally controlled conditions.

Here, we use the cestode *Schistocephalus solidus* and its copepod host to compare the effect of single with multiple infections on host behavior. We study especially the outcome of a conflict between coinfecting parasites over host manipulation using laboratory-bred, hence parasite-free, hosts. *Schistocephalus solidus* has a three-host life cycle. From the first intermediate host, a copepod, the parasite is trophically transmitted to the next host, the three-spined stickleback, a fish, which has to be subsequently consumed by a bird for the parasite to complete its life cycle (Clarke 1954; Dubinina 1980). In the copepod, *S. solidus* initially reduces the activity of its host (Hammerschmidt et al. 2009) and thus the host's risk of being preyed upon (Weinreich et al. 2013). Once ready for transmission, *S. solidus* switches the direction of host manipulation to increasing its host's activity (Wedekind and Milinski 1996; Hammerschmidt et al. 2009), risk taking (Jakobsen and Wedekind 1998), and predation susceptibility (Wedekind and Milinski 1996). We show that two coinfecting parasites (1) with the same aim cooperate, but (2) with a conflict of interest sabotage each other's manipulation.

## Materials and Methods

### HOSTS

Copepods (*Macrocyclops albidus*) came from a laboratory culture originated from populations from the “Neustaedter Binnenwasser,” northern Germany, where sticklebacks are naturally infected by *S. solidus*. One day prior to the first exposure to the parasites, copepods were filtered from their home tanks and each individual copepod was transferred to a well in a 24-well plate with about 1 mL of water. To reduce variation with regard to the host, only adult male copepods were used. We used a total of 1992 copepods in two separate experiments (1248 in experiment 1, 744 in experiment 2). During both experiments, copepods were fed with five *Artemia* sp. nautili and the wells cleaned if necessary every other day (always a day on which no infections or behavioral recordings took place, i.e., day 1, 3, 5, 8, 10, 12, 14, 16, 18, 20, and 22 after the first infection). The copepods were kept at 18°C in a 16h/8h light/dark cycle. Because our behavioral essays included the reaction to disturbance, we took care to avoid other disturbances to prevent previous habituation to our test.

### PARASITES

*Schistocephalus solidus* were bred in an in vitro system in the laboratory (Smyth 1946; Wedekind 1997). We used offspring from parasites dissected from naturally infected fish caught at the “Neustaedter Binnenwasser,” northern Germany. Currently, infection rates in sticklebacks are low (below 1%, Hafer unpubl. data) in this population but had been above 30% some years ago (Kalbe and Milinski unpubl. data). Because of a considerable time interval between our two experiments, we used different parasite families in each experiment. Eggs were stored in the fridge (4°C) until use. Prior to infection, they were incubated for three weeks at 20°C in the dark and then exposed to light overnight to induce the coracidia to hatch (Dubinina 1980).

### INFECTIONS

**Figure 1:**
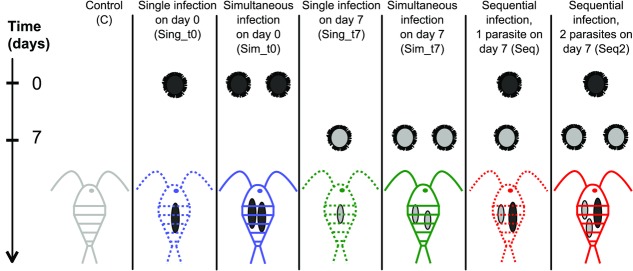
Timing of infections with different numbers of parasites to achieve the required treatments. Copepods were exposed to zero, one, or two *Schistocephalus solidus* on day 0 (t0) and on day 7 (t7) in a manner that resulted in seven different treatments.

Infections took place at two different time points, the day after the copepods had been distributed onto the plates (day 0) and one week later (day 7). For experiment 1, each copepod was exposed to zero, one, or two parasites on each of these days in a manner that resulted in six different treatments (Fig.1): Unexposed controls (C), singly exposed to one parasite on day 0 (Sing_t0), simultaneously exposed to two parasites on day 0 (Sim_t0), singly exposed to one parasite on day 7 (Sing_t7), simultaneously exposed to two parasites on day 7 (Sim_t7), and sequentially exposed to two parasites, that is, exposed to a single parasite each on day 0 and day 7 (Seq) (Fig.1). To account for the size differences between the parasites from the first and second infection in sequential infections and potentially resulting differences in how strongly they were able to manipulate, we conducted an additional experiment (experiment 2) in which we infected copepods with one parasite on day 0 and with either zero (Sing_t0), one (Seq), or two (Seq) additional parasites on day 7. We were unable to measure parasite size within our experiment because that would have exposed the copepods to substantial stress. However, we did so during a preliminary study to confirm that two noninfective parasites could make up about the same volume as one infective one (Supporting Information Results 2, Fig. S3). Several copepods were not exposed at all to obtain uninfected controls (C) or only on day 7 to verify the timing of manipulation by a noninfective parasite when alone (Sing_t7) (Fig.1).

We used three (experiment 1) or four (experiment 2) different parasite families to infect copepods and if a copepod received more than one parasite, they always originated from different families. Treatments were evenly distributed over all plates and randomly arranged on each plate.

To verify that an infection had occurred, we placed each copepod under a microscope. Male copepods are transparent making it possible to see any parasite within the living copepod. This took place only after all behavioral recordings had been completed in order not to interfere with copepod behavior (experiment 1: day 23 and 24, experiment 2: day 21). By this time, we expected any parasite to have become infective to the subsequent host. Unfortunately that did not allow us to document the development of individual parasites. However, previous studies found that the rate of development shows little flexibility. Within 11 days postinfection, more than 80% of parasites developed a cercomer, which is a good indication that the parasite will soon become infective to the next host (Benesh 2010a,b). Copepods that died before day 24 but after day 13 (experiment 1) or day 19 (experiment 2) were checked in the same manner, although a determination of the infection status was only possible in those copepods that had not yet started to decay. Their behavior was only used until three days prior to their death to exclude the behavior of dying copepods from the dataset. We only included copepods in the subsequent analysis that were correctly infected according to their treatment by all parasites they had been exposed to. That way we could exclude that differences between treatments were caused by initial differences between copepods, which were also responsible for whether a copepod, exposed to a coracidium, was indeed infected by this coracidium (or altered by the effect of a failed infection, which cannot be excluded in mass infections). This resulted in a total of 147 copepods for experiment 1 (C: 41, Sing_t0: 25, Sing_t7: 27, Sim_t0: 11, Sim_t7: 25, Seq: 18) that could be analyzed. Of those copepods used for the analysis, 25 died during the experiment. For experiment 2, we could obtain data from a total of 111 copepods, one of which died during the experiment (C: 20, Sing_t0: 25, Sing_t7: 22, Seq: 28, Seq2: 26).

### BEHAVIORAL RECORDINGS AND ANALYSIS

Copepod behavior was recorded by carefully placing a 24-well plate with copepods on an apparatus that dropped it by 3 mm in a standardized manner to simulate a failed predator attack (Hammerschmidt et al. 2009). After such a predator attack, the predator is likely to be still present for some time and likely to try attacking the copepod again. Hence, the period after the simulated predator attack should be perceived as one of increased predation risk by the copepod. Under these circumstances, predation avoidance should be especially crucial and predation enhancement most efficient and we would hence expect them to be strongest. The drop took place after the plate had been on the apparatus for 1 min. Starting just before the drop, we video recorded the copepods on the plate for 15 min with a camera (Panasonic Super DynamicWV-BP550 Panasonic Corporation, Osaka, Japan). Behavioral recordings took place every other day starting on day 9 until day 23 (experiment 1) or day 21 (experiment 2), always on the day when copepods were not fed.

We analyzed copepod behavior (i.e., activity) during 1 min right after the simulated predator attack when, following a movement to escape predation, copepods should reduce activity to avoid detection by a potential predator (starting 10 sec after the simulated predator attack to avoid the initial escape reaction, see Hammerschmidt et al. 2009) and at the end of the recorded period (i.e., between 14 and 15 min after the simulated predator attack), when the copepods could be assumed to have recovered from the simulated predator attack. Using the manual tracking plugin within image J (Rasband 2008), we recorded whether each copepod moved within each 2-sec interval. All analyses were done blindly with regard to the copepod's treatment.

### STATISTICAL ANALYSIS

Data were analyzed in R (R Development Core Team 2010) using generalized linear mixed models in the lme4 package (Bates et al. 2014). We used copepod identity as random effects including the day after the first infection to account for the presence of intra individual variation between days and the period in the recording to account for intra individual variation over time. We fitted a model for the time moved as response variable using binomial distribution to account for the distribution of the data. We further included both the day after the first infection and period in the recording (i.e., after a simulated predator attack vs. after a recovery period). We stepwise added the treatment and all its interactions with day and the period in the recording to the model. Separate models were fitted for experiment 1 and 2 because not all treatments were present in both. Subsequently, we performed likelihood ratio tests to compare models and find those that gave the best fit. A model was accepted if it was significantly better than a less complex model at explaining the data. The complete outputs of the models are presented in Table S1.

For each treatment and period in the recording (i.e., after a simulated predator attack/ after a recovery period), we performed a separate Tukey's test using general linear hypotheses within the multcomp package in R (Hothorn et al. 2008) to determine between which consecutive days significant chances in host behavior took place. The same was done for each day and period in the recording to find out when and between which treatments differences occurred. Only those statistics directly relevant for our question are reported in the Results. For a complete overview of the statistical results, refer to Tables S2–S5.

## Results

The behavior of the copepod hosts was significantly influenced by the three-way interaction between the parasite treatment they received, the day *post infection* on which the recording took place, and the period in the recording (i.e., after a simulated predator attack or after a recovery period; *P* < 0.001, see Table S1 for further information). Hence, we conducted Tukey's HSD tests for multiple comparisons for each treatment or day and period in the recording to determine when copepod activity changed significantly between days within each treatment and when and between which treatments significant differences occurred. Only *P*-values for the multiple comparisons are reported here. Please refer to Tables S2–S5 for exact statistical outputs. Here, we present only the results we observed directly after a simulated predator attack, because results once the copepods had had time to recover were similar although less pronounced. They are presented in Supporting Information Results 1 and Figures S1, S2.

### CHANGE OF COPEPOD HOST ACTIVITY OVER TIME

**Figure 2:**
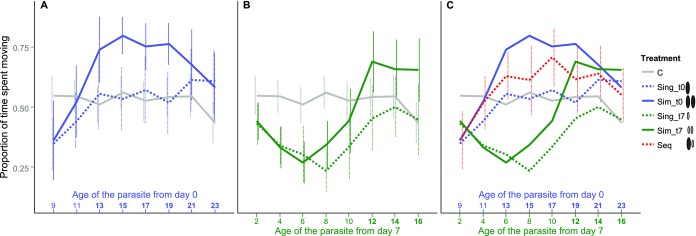
Activity of copepods according to treatment, right after a simulated predator attack. Error bars indicate 95% CI. Bold numbers on the x-axis indicate that a parasite of that age was infective. (A) Copepods infected on day 0, (B) copepods infected on day 7, (C) all treatments. Error bars from the treatments already presented in (A) and (B) have been omitted for better readability. C, unexposed control copepods; Sing_t0, copepods singly infected with one parasite on day 0; Sim_t0, copepods simultaneously infected with two parasites on day 0; Sing_t7, copepods singly infected with one parasite on day 7; Sim_t7, copepods simultaneously infected with two parasites on day 7; Seq, copepods sequentially infected with two parasites, one each on day 0 plus day 7.

We measured the activity of the copepods right after a simulated predator attack. We expected that shortly after infection, the parasite would start manipulating its copepod host by decreasing its activity and thus its predation risk (predation suppression) because it would be too early for the parasite to be transmitted to the next host. Once the parasite has reached infectivity for the next host, copepod activity should be increased (predation enhancement) as shown previously (Hammerschmidt et al. 2009). The initial decrease in host activity has been studied before (Hammerschmidt et al. 2009), so that we started recording of host behavior only from day 9 in the experiment, that is, nine days after the first infection that took place on day 0, just before the switch in host manipulation is expected to occur in copepods singly infected on day 0 (dashed blue line in Fig.2). After the parasites had become infective, copepod activity increased as expected between day 9 and 11, and 11 and 13 (*P* < 0.001). Those copepods that were singly infected by one parasite on day 7 (dashed green line in Fig.2) displayed the expected delay and showed initial decrease in activity (predation suppression) between day 9 and 11 (i.e., when the parasite was between two- and four-day old, *P* = 0.003). They also displayed the expected increase in activity after the parasite reached infectivity (predation enhancement, *P* < 0.001) at the same time *post infection* when copepods singly infected on day 0 showed increased activity (between day 9 and 13 *post infection*, i.e., between day 15 and 19 in the experiment). Note that parasites administered to copepods on day 0 have always been for seven days longer in the copepod than parasites infecting copepods on day 7 when their behavior is recorded (Fig.1). The control group (unexposed copepods) did not show any significant changes in host activity throughout the course of the experiment (gray line in Fig.2, *P* > 0.5).

We found significant differences between the behavior of control copepods (gray line) and copepods singly infected at either infection time point (day 0 or day 7, dashed blue and green lines, respectively, *P* < 0.03) during expected predation suppression (on day 9 for copepods infected on day 0 and between day 11 and 13 in the experiment for copepods infected on day 7, i.e., between day 4 and 9 *post infection*). After host activity had increased again (i.e., once the parasite was at least 10 days old), no differences between control copepods and singly infected copepods were significant (*P* > 0.2). Hammerschmidt et al. (2009) observed both predation suppression before the parasite reached infectivity and predation enhancement after it had reached infectivity. Although we can confirm the existence of predation suppression and a switch in host manipulation to enhance predation, we did not observe actual predation enhancement beyond the level of control copepods. However, Hammerschmidt et al. (2009) found predation enhancement especially when measuring the time copepods needed to recover from a simulated predator attack, and much less with regard to the copepods’ activity, which is what we focused on in this study. Thus, after generally having confirmed previous findings, we can test for synergy and conflict over host manipulation in experimental double infections.

### POTENTIAL SYNERGY OF PARASITES IN SIMULTANEOUS DOUBLE INFECTIONS

We expected that in copepods that harbored two parasites of the same age and hence the same interest, the parasites should strengthen each other's manipulation. Such copepods behaved similarly compared to those infected with just one parasite at the same time point: Copepods infected with two parasites on day 0 (continuous blue line in Fig.2) significantly increased their activity between day 9 and 13, that is, when their parasites became infective (*P* < 0.001). In copepods infected with two parasites on day 7 (continuous green line), the onset of manipulation was marked by a significant decrease in host activity between day 9 and 11 (*P* < 0.001), which was followed by a significant increase between day 15 and 19 (*P* < 0.004), that is, when also these parasites had reached infectivity. Behavior of copepods singly or simultaneously infected on day 7 was significantly different from unexposed control copepods during expected predation suppression, that is, between day 11 and 15 (*P* < 0.02), but not on any other day (*P* > 0.07). Copepods simultaneously infected on day 0 tended to be more active than controls on day 15 (*P* = 0.059), but not on any other day (*P* > 0.09).

We found indeed evidence for synergy effects during predation enhancement: simultaneously infected copepods had a significantly higher activity than singly infected copepods from the same infection time point (copepods infected on day 0, day 15: *P* = 0.049, blue lines; copepods infected on day 7, day 19: *P* = 0.026, green lines). These differences were significant only after the parasites had reached infectivity, that is, during predation enhancement, but not before (*P* > 0.6), that is, during predation suppression.

### THE OUTCOME OF A CONFLICT BETWEEN PARASITES OVER HOST MANIPULATION

If one parasite is infective (and hence should enhance its host's predation risk) and the other one is noninfective (and we therefore expect predation suppression), there is potential for a conflict over the direction of host manipulation between the two parasites. We confirmed that such a conflict exists by comparing copepods singly or simultaneously infected on day 0 to those infected with the same number of parasites on day 7: Copepods singly infected on day 0 (dashed blue line) were significantly more active than copepods singly infected on day 7 (dashed green line) from day 13 to day 17 (*P* < 0.03). The same was true for simultaneously infected copepods (continuous blue and green line; *P* < 0.001). We did not observe any significant differences between parasites infected on day 0 or day 7 with the same number of parasites on any other day (*P* > 0.1). These results defined the time interval during which parasites that infected the copepod on day 0 were already infective and inducing predation enhancement and parasites that infected the copepod on day 7 and were not infective yet, and induced predation suppression, indicating the time window of conflict. If parasites from either infection time point (day 0 or day 7) have about equal strength of manipulation, we would expect that the behavior of copepods with one infective parasite (i.e., one parasite that infected the copepod on day 0) plus one noninfective parasite (i.e., one parasite that infected the copepod on day 7) (dashed red line) is intermediate between that of copepods with parasites from only one infection time point (either day 0 or day 7, blue and green lines) during the window of conflict, that is, the dashed red line should be between the blue and the green lines in Fig.2.

During the period of conflict over host manipulation, copepods that were sequentially infected with one parasite on day 0 plus one on day 7 (dashed red line in Fig.2) differed significantly only from copepods singly or simultaneously infected on day 7 (green lines, day 13–17: *P* < 0.050). Throughout the experiment, those copepods sequentially infected on day 0 plus on day 7 (dashed red line) never differed significantly from copepods singly or simultaneously infected on day 0 (blue lines) (*P* > 0.4). Thus, the noninfective parasite that infected the copepod on day 7 has no detectable effect in sequential infections with an already infective parasite administered to the copepod on day 0.

Consequently, changes over time in the behavior of copepods sequentially infected on day 0 and day 7 (dashed red line) mostly resembled copepods infected only on day 0 (blue lines): Copepod activity increased from one day to the next when the parasite administered to the copepod on day 0 became infective to the next host between day 9 and 13 *post infection* (dashed red line, *P* < 0.05). However, unlike in copepods only infected on day 0 (blue lines) copepod activity significantly increased further between day 15 and 17 (*P* = 0.015). This later increase occurred at the time when the parasite administered on day 7 should be reaching infectivity (between day 8 and 10 *post infection*). At this time, the conflict between the two parasites vanishes and synergy may begin. This fits well with the fact that parasites in simultaneously infected copepods enhance each other's manipulation once both parasites have reached infectivity (see above).

In the most parsimonious mechanistic scenario where both disagreeing parasites continue to manipulate as if alone, we had expected the outcome of this conflict to be somewhat intermediate. This is not the case. Rather, the parasite administered on day 0 wins the conflict, making its host behave indistinguishably from a host infected only on day 0 and not on day 7. Thus, the infective parasite that infected the copepod on day 0 suppresses the manipulation by the noninfective parasite that infected the copepod on day 7.

### EQUAL POTENTIAL STRENGTH OF PARASITES THAT ARE AT A CONFLICT OVER HOST MANIPULATION

**Figure 3:**
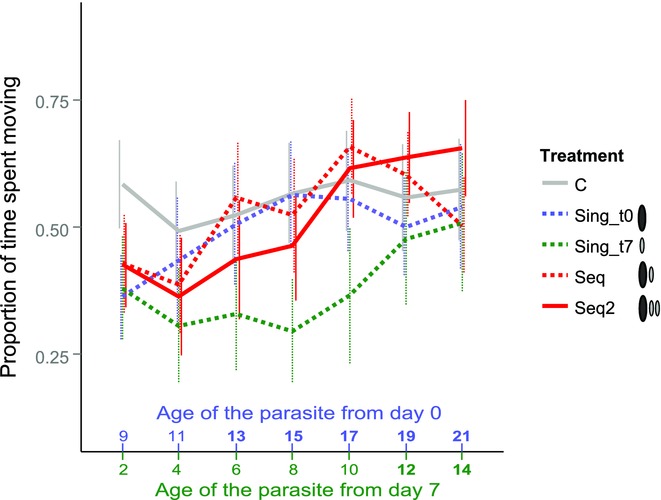
Activity of copepods according to treatment, right after a simulated predator attack. Error bars indicate 95% CI. Bold numbers on the x-axis indicate that a parasite of that age was infective. C, unexposed control copepods; Sing_t0, copepods singly infected with one parasite on day 0; Sing_t7, copepods singly infected with one parasite on day 7; Seq, copepods sequentially infected with two parasites, one each on day 0 plus day 7; Seq2, copepods sequentially infected with three parasites, one on day 0 plus two on day 7.

The missing effect of the noninfective parasite in copepods sequentially infected on day 0 plus day 7 could be due to a size difference between the infective parasite from day 0 and the noninfective parasite from day 7. Parasites administered on day 0 were always larger than those administered on day 7 (Supporting Information Results 2, Fig. S3). This could help the infective parasite from day 0 to overpower the noninfective parasite from day 7. Hence, in a separate experiment, we compared copepods sequentially infected with one parasite on day 0 plus two on day 7 (continuous red line in Fig.3) to copepods infected only on day 0 (dashed blue line in Fig.3), only on day 7 (dashed green line in Fig.3), and copepods sequentially infected with one parasite on day 0 plus one on day 7 (dashed red line in Fig.3). Copepods sequentially infected with one parasite on day 0 plus two on day 7 (continuous red line) were never significantly different from copepods sequentially infected with one parasite on day 0 plus one on day 7 (dashed red line, *P* > 0.1). So, combined volumes of two noninfective parasites from day 7 did not make a detectable difference to only one noninfective parasite from day 7 in sequentially infected copepods already infected by a parasite on day 0. Additionally, copepods sequentially infected with one parasite on day 0 plus two on day 7 (continuous red line) never differed significantly from copepods only infected on day 0 (dashed blue line, *P* > 0.3). They did, however, differ from copepods infected only on day 7 (dashed green line) between day 13 and 17. This was only a trend on day 13 (*P* = 0.056), but significant thereafter (*P* < 0.01). Thus, one noninfective parasite from day 7 alone in a copepod has a stronger effect than even two noninfective parasites from day 7 if their host was previously infected by one parasite on day 0 that is now infective.

In conclusion, we find no significant effect of the second noninfective parasite from day 7 in copepods sequentially infected on day 0 plus day 7. The two parasites from day 7 have about the same volume as the one parasite from day 0 (Supporting Information Results 2, Fig. S3). Thus, if a copepod is infected by two noninfective parasites, both together should have the strength to suppress the manipulated activity of the host to reduce its predation risk to an intermediate level. Instead, it is still the single infective one that wins the conflict.

## Discussion

A noninfective *S. solidus* parasite should prevent its current copepod host from being eaten by the next host, a stickleback fish—it dies when transmitted too early, whereas an infective parasite should increase its copepod's predation risk—it continues its life cycle when transmitted. This is what both achieve when alone in a copepod (Wedekind and Milinski 1996; Hammerschmidt et al. 2009; Benesh 2010b; Weinreich et al. 2013). When an infective and a noninfective *S. solidus* parasite share the same copepod, they are at a conflict over the direction of host manipulation. The infective parasite clearly wins the conflict, whereas the noninfective one seems to have no effect on host behavior at all. This is true not only when the predation risk is high but also after it has returned to normal (see Supporting Information Results 1).

Why does the noninfective parasite fail to reduce manipulation by the infective one, which is potentially disastrous for its fitness? When each parasite is alone, the noninfective one has a large effect on host behavior, whereas the effect of aninfective one was not even measurable in our study. In coinfections, the infective parasite due to its larger volume could have an advantage and be able to produce more host manipulation. We controlled for this by using also two noninfective parasites to allow them to gain about the same volume (and an even larger surface) than the infective parasite. They remained unable to have a distinguishable effect on host behavior. This seems to be the case even if three noninfective parasites are used (Supporting Information Results 2, Fig. S4). Parasites can maximize their fitness by being transmitted to the next host at an optimal time point (Hammerschmidt et al. 2009; Parker et al. 2009). Additionally, by infecting the subsequent stickleback host before the younger coinfecting parasite is ready for transmission, the infective parasite can exclude competition in the subsequent host. Nevertheless, a parasite transmitted later than optimal may still complete its life cycle and reproduce successfully. In contrast, a parasite that is transmitted too early will always fail to infect a fish, achieving a fitness of zero (Hammerschmidt et al. 2009).

Clearly, the noninfective parasite has nothing to gain from facilitating the transmission of the infective one by reducing its own manipulation. In nature it is unlikely, in our experiment we excluded that two parasite larvae independently consumed by a copepod are close kin. Reallocating energy saved from not manipulating to faster development is no better option. Benesh (2010b) found no significant correlation between host manipulation and growth and development of individual parasites. It is plausible that the infective parasite wins the conflict over host manipulation if it actively suppresses the manipulation exerted by the noninfective one: it would be transmitted at an optimal time point. To our knowledge, our findings present the first clear evidence that one parasite successfully sabotages the host manipulating of a coinfecting parasite under strictly experimental conditions.

Any opposition of the noninfective parasite to being suppressed will not be favored by selection if the mortality of a copepod already harboring an infective parasite is so high in nature that the second parasite will not reach infectivity. A similar scenario with one side having no fitness gain from opposing to being exploited are the slaves that are stolen as pupae by slave maker ants from foreign nests and brought to their own nest where the slaves raise the slave maker queen's offspring. Usually, workers win the conflict with their queen over the sex ratio of the next generation, which is 3:1 in favor of female reproductives in ants (Trivers and Hare 1976). Having no fitness in the slave maker nest anyway, ant slaves do not gain from opposing to manipulation by the slave maker queen, thus a mutant would not pass on any genes, they produce a 1:1 sex ratio completely in line with the slave maker queen's interests (Nonacs 1986).

If two parasites sharing a host have the same interests, either predation suppression or enhancement, both may profit from amplifying each other's manipulation and/or sharing potential costs. We find that after both parasites reached infectivity, they increased host activity more than a single one does. This agrees with findings of an observational study (Urdal et al. 1995). Wedekind and Milinski (1996) found a positive correlation between the activity of both infected and uninfected copepods and predation susceptibility. Thus, an additional increase in host activity through manipulation would lead to predation enhancement. Two noninfective parasites did, however, not amplify each other's manipulation in the present study, nor in a prior observational study (Urdal et al. 1995). Has the parasite an optimal level of manipulation it attempts to reach or does any increase in manipulation convey a fitness benefit? This might well differ before and after reaching infectivity. During predation suppression, decreasing host activity below a certain level might be disadvantageous for the parasite. It might prevent its host from consuming enough energy to allow the parasite to ever reach infectivity, especially when two parasites compete for energy. Two *S. solidus* that share a copepod host grow to a smaller size (Michaud et al. 2006). Accordingly, the number of noninfective parasites has no significant effect on predation susceptibility of copepods infected with noninfective *S. solidus* (Weinreich et al. 2013). However, noninfective parasites may share the potential cost of manipulation.

For a parasite to evolve to either cooperate with a conspecific or to sabotage its manipulation, selection pressures have to be high enough. They will depend largely on the likelihood for a parasite to encounter such a conspecific (Rigaud and Haine 2005). Despite a very low prevalence of *S. solidus* in its copepod host, double infections do occur, albeit rarely (Zander et al. 1994). This seems to be a general pattern for cestode-copepod systems (e.g., Zander et al. 1994; Pasternak et al. 1995; Hanzelová and Gerdeaux 2003). Despite those usually low infection rates, there is some evidence that *S. solidus* has evolved strategies to deal with the presence of conspecifics in its copepod host in addition to the present study (Wedekind 1997; Michaud et al. 2006). In both, the second intermediate fish host (e.g., Arme et al. 1967; Heins et al. 2002) and the definite bird host (e.g., Chubb et al. 1995), very high infection intensities can occur. It would hence be plausible that *S. solidus* prevalence in copepods is strongly increased locally, for example, underneath roosting trees were highly infected birds defecate (Michaud et al. 2006). The frequency of coinfections typically correlates positively with parasite prevalence (Louhi et al. 2013).

Naturally, the results of our study raise questions about the underlying mechanisms. A parsimonious mechanistic explanation would require active manipulation only for one type of manipulation (i.e., predation suppression or predation enhancement) and/or the switch in host manipulation. Predation suppression via decreased activity prior to reaching infectivity could be a stress response to infection (e.g., Poulin 1995, 2010; Thomas et al. 2005; Moore 2013). However, doubled stress by two noninfective parasites had no additional effect. Moreover, such a stress response would have to be switched off precisely when the parasite becomes infective. The subsequent predation enhancement (i.e., increased activity) would not require actual manipulation. It could be due to increased energy drain inevitably caused by growing parasite(s) (Milinski 1990). Animals optimally trade-off feeding and avoiding predation, shifting the compromise to the higher need (Milinski and Heller 1978). Higher energy drain would lead to accepting higher predation risk. Naturally, this effect of energy drain would also be caused by noninfective parasites and needs to be suppressed or counterbalanced. Sabotage by the infective parasite of the noninfective one's manipulation could be done with the same mechanism with which the infective parasite switches from predation suppression to predation enhancement. This hypothesis implies changing a hormone to a pheromone and probably producing the substance in higher quantity. Further studies of the mechanisms underlying host manipulation will be necessary to understand how one parasite manages to sabotage another parasite's manipulation.

One very common mechanism of host manipulation seems to be the modification of neuromodulatory systems that are closely linked to the immune system with which parasites have to cope in any case. Accordingly, parasites could exploit this link to manipulate host behavior (Adamo 2002; Helluy 2013; Lafferty and Shaw 2013). Especially in sequential coinfections, any effect of the first parasite would be likely to affect the interaction of the second parasite with the immune system. The initial establishment seems to be the crucial part of host–parasite interactions in *S. solidus* infections in copepods (van der Veen and Kurtz 2002). Prior infection with a closely related *S. solidus* reduces susceptibility to a second parasite (Kurtz and Franz 2003), but during simultaneous infections, the chances for a single parasite to establish increase with increasing number of parasites administered (Wedekind 1997). Parasites can be lost for a few days after infections, but this seems to be due to intrinsic mortality rather than the host's immune system or within host competition (van der Veen and Kurtz 2002). Unfortunately, we do not know if host manipulation in *S. solidus* in its copepod host is in any way linked to the parasite's interaction with the host's immune system.

Parasites agreeing or disagreeing over whether and how their shared host should be manipulated are expected to be ubiquitous in nature. Even human infectious diseases manipulate their host, for example, human toxoplasmosis with a worldwide prevalence of about 30% is supposed to permanently manipulate the behavior of infected people (Flegr 2013). Its manipulation could be altered by coinfecting parasites (De Queiroz et al. 2013). Our article presents a proof of principal that one parasite can impact and even neutralize the manipulation by another parasite.

## DATA ARCHIVING

The doi for our data is 10.5061/dryad.3s7r7.

## Supporting Information

Disclaimer: Supplementary materials have been peer-reviewed but not copyedited.

**Figure S1**. Activity (i.e., proportion of time spent moving) of copepods according to treatment, after a recovery period.

**Figure S2**. Activity (i.e., proportion of time spent moving) of copepods according to treatment, after a recovery period.

**Figure S3**. Relative combined size of day-7 parasites depending on the number of day-7 parasites per copepod.

**Figure S4**. Activity (i.e. proportion of time spent moving) of copepods according to treatment, after a simulated predation attack (A) and after a recovery period (B).

**Results 1**. Results after a recovery period.

**Results 2**. Additional confirmation of the effect of the number of non-infective parasites from day 7

**Table S1**. Outcome of likelihood ratio tests.

**Table S2**. Outcome of multiple comparisons between days for each treatment and period in the recording (i.e., after a simulated predation attack vs. after a recovery period).

**Table S3**. Outcome of multiple comparisons between treatments for each day and period in the recording (i.e., after a simulated predation attack vs. after a recovery period).

**Table S4**. Outcome of multiple comparisons between days for each treatment and period in the recording (i.e., after a simulated predation attack vs. after a recovery period).

**Table S5**. Outcome of multiple comparisons between treatments for each day and period in the recording (i.e., after a simulated predation attack vs. after a recovery period).

**Table S6**. Outcome of likelihood ratio tests.

**Table S7**. Outcome of multiple comparisons between treatments for each day and period in the recording (i.e., after a simulated predation attack (A) vs. after a recovery period (B)).
