# Supplementary material for: When parasites disagree: Evidence for parasite-induced sabotage of host manipulation
Source: Evolution. 2015 Mar 10;69(3):611–20. doi: 10.1111/evo.12612 (PMC4409835; doi:10.1111/evo.12612)
Supplement: Supplementary file 1 — Figure S1. Activity (i.e., proportion of time spent moving) of copepods according to treatment, after a recovery period. [file evo0069-0611-sd1.doc]

Figure S1: Activity (i.e. proportion of time spent moving) of copepods according to treatment, after a recovery period. Error bars indicate 95% CI. Bold numbers on the X-axis indicated that a parasite of that age was infective. A: Copepods infected on day 0, B: Copepods infected on day 7, C: All treatments. Error bars from the treatments already presented in A and B have been omitted for better readability. C: uninfected control copepods (*n* = 41), Sing_t0: copepods singly infected with one parasite on day 0 (*n* = 25), Sim_t0: copepods simultaneously infected with two parasites on day 0 (*n* = 11), Sing_t7: copepods singly infected with one parasite on day 7 (*n* = 27), Sim_t7: copepods simultaneously infected with two parasites on day 7 (*n* = 25), Seq: copepods sequentially infected with two parasites, one each on day 0 plus day 7 (*n* = 18).
