# Supplementary material for: When parasites disagree: Evidence for parasite-induced sabotage of host manipulation
Source: Evolution. 2015 Mar 10;69(3):611–20. doi: 10.1111/evo.12612 (PMC4409835; doi:10.1111/evo.12612)
Supplement: Supplementary file 2 — Figure S2. Activity (i.e., proportion of time spent moving) of copepods according to treatment, after a recovery period. [file evo0069-0611-sd2.doc]

Figure S2: Activity (i.e. proportion of time spent moving) of copepods according to treatment, after a recovery period. Error bars indicate 95% CI. Bold numbers on the X-axis indicated that a parasite of that age was infective. C: uninfected control copepods (*n* = 20), Sing_t0: copepods singly infected with one parasite on day 0 (*n* = 25), Sing_t7: copepods singly infected with one parasite on day 7 (*n* = 22), Seq: copepods sequentially infected with two parasites, one each on day 0 plus day 7 (*n* = 28), Seq2: copepods sequentially infected with three parasites, one on day 0 plus two on day 7 (*n* = 26).
