# Supplementary material for: When parasites disagree: Evidence for parasite-induced sabotage of host manipulation
Source: Evolution. 2015 Mar 10;69(3):611–20. doi: 10.1111/evo.12612 (PMC4409835; doi:10.1111/evo.12612)
Supplement: Supplementary file 3 — Figure S3. Relative combined size of day-7 parasites depending on the number of day-7 parasites per copepod. [file evo0069-0611-sd3.doc]

Figure S3: Relative combined size of day-7 parasites depending on the number of day 7 parasites per copepod. Error bars indicate 95% CI. The horizontal black line indicates equal size of the parasite from day 0 and all parasites from day 7 within that copepod. Each copepod was infected by one parasite on day 0 and (n=19), 2(n=16) or 3 (n=5) parasites on day 7.
