# Supplementary material for: When parasites disagree: Evidence for parasite-induced sabotage of host manipulation
Source: Evolution. 2015 Mar 10;69(3):611–20. doi: 10.1111/evo.12612 (PMC4409835; doi:10.1111/evo.12612)
Supplement: Supplementary file 4 — Figure S4. Activity (i.e. proportion of time spent moving) of copepods according to treatment, after a simulated predation attack (A) and after a recovery period (B). [file evo0069-0611-sd4.doc]

Figure S4: Activity (i.e. proportion of time spent moving) of copepods according to treatment, after a simulated predation attack (A) and after a recovery period (B). Error bars indicate 95% CI. Each copepod was infected by one parasite on day 0 and 0 (n=22-22), 1(n=33-39), 2(n=23-30) or 3 (n=7-9) parasites on day 7.
