# Supplementary material for: When parasites disagree: Evidence for parasite-induced sabotage of host manipulation
Source: Evolution. 2015 Mar 10;69(3):611–20. doi: 10.1111/evo.12612 (PMC4409835; doi:10.1111/evo.12612)
Supplement: Supplementary file 5 — Results 1. Results after a recovery period. [file evo0069-0611-sd5.doc]

# Results SI1: Results after a recovery period

### Change of copepod host activity over time

After copepods had had time to recover, copepods singly infected on day 0 (Fig. S1, dashed blue line), increased their activity between day 11 and 13 (p=0.009), but not before (p>0.5). Copepods singly infected on day 7 (Fig. S1, dashed green line) initially decreased their activity as host manipulation set in between day 9 and 11 in the experiment (p<0.001). They increased their activity again at the same time *post infection* as copepods infected on fay 0, i.e. between 11 and 13 days *post infection*, between day 17 and 19 in the experiment (p<0.001). Unexposed control copepods, however, also increased their activity between day 11 and 13 (p<0.001) but decreased it between day 17 and 19 (p=0.043). Before the parasite reached infectivity (predation suppression) singly-infected copepods were always significantly different from control copepods (p<0.03). Copepods singly on day 7 continued to be significantly less active than control copepods throughout the experiment (P<0.001) except on day 19 (p=0.331).

### Potential synergy of parasites in simultaneous double infections

Like copepods singly infected on day 0, copepods infected with two parasites on day 0 (Fig. S1, continuous blue line) significantly increased their activity between day 11 and 13 (p<0.001). In those infected with two parasites on day 7 (Error: Reference source not found, continuous green line), the onset of manipulation was marked by a significant decrease in host activity between day 9 and 11 (p<0.001) and the switch from predation suppression to predation enhancement by a significant increase between day 17 and 19 (p<0.002). Copepods simultaneously infected on day 0 never significantly differed from controls (p>0.3). Copepods simultaneously on day 7 were significantly less active than controls during predation suppression, i.e. between day 11 and 17 (p<0.2). We never observed any significant differences between singly-infected copepods and simultaneously-infected copepods from the same infection time point (p>0.07).

### The outcome of a conflict between parasites over host manipulation

The conflict we observed immediately after a simulated predation attack was also observable after a recovery period. On day 15, copepods singly infected on day 0 were significantly more active than those singly infected on day 7 (p=0.003). The same was true when comparing simultaneously-infected copepods on day 15 and 17 (p<0.04). No other significant differences occurred for those comparisons (p>0.06). Sequentially-infected copepods (i.e. infected with one parasite each on day 0 plus on day 7, Fig. S1 dashed red line), however, never differed from simultaneously-infected copepods (p>0.1). They were never significantly different from copepods singly infected on day 0, either (p>0.7), but did differ from those singly infected on day 7 on day 17 and day 21 (p<0.03), though not on any other day (p>0.1). Hence, there is less evidence for a conflict over host manipulation after a recovery period, but if anything it still seems to be won by the infective parasite from day 0.

### Equal potential strength of parasites that are at a conflict over host manipulation

We never observed any significant differences between copepods singly infected on day 0 (Fig. S2 dashed blue line) and copepods sequentially infected with one parasite on day 0 and one (Fig. S2, dashed red line) or two (Fig. S2, continuous red line) on day 7 (p>0.2). Copepods sequentially infected with one parasite on day 0 plus two on day 7 did differ significantly from those only infected on day 7 (Fig. S2, dashed green line) on day 13 (p=0.007), but on no other day (p>0.08). Just like after a simulated predation attack, we find no clear evidence that increasing parasite number changes the outcome of the conflict over host manipulation.
