# Supplementary material for: When parasites disagree: Evidence for parasite-induced sabotage of host manipulation
Source: Evolution. 2015 Mar 10;69(3):611–20. doi: 10.1111/evo.12612 (PMC4409835; doi:10.1111/evo.12612)
Supplement: Supplementary file 6 — Results 2. Additional confirmation of the effect of the number of non-infective parasites from day 7 [file evo0069-0611-sd6.doc]

# Results SI2: Additional confirmation of the effect of the number of non-infective parasites from day 7

## Material and methods

Copepods were exposed to one *S. solidus* on day 0 and 1 or 5 on day 7 in the same manner described in the main paper. The parasite administered on day 0 always originated from a different parasite family than the parasite(s) administered on day 7, but if 5 parasites were used on day 7, they resulted from the same family. Unlike in the main experiment we used any copepod infected by one parasite on day 0 and any number of day 7 parasites, resulting in 103 copepods in 4 different treatments infected with one parasite on day 0 and a variable number of parasites on day 7 (0 parasites on day 7: 25, 1 parasite on day 7: 39, 2 parasites on day 7: 30, 3 parasites on day 7: 9). Only one copepod was infected by more than 3 parasites on day 7 and excluded from analysis.

Parasites and copepods stemmed from the same population and were maintained in the same manner described in the main paper. We checked copepods for infection on day 15, i.e. when parasites from day 0 were 15 and parasites from day 7 were 8 days old and non-infective. We measured parasite size on day 16 (parasites from day 7 were 9 days old and hence still non-infective) in copepods infected with one parasite on day 0 and at least one on day 7. It is possible to do so in the living copepod (e.g. Wedekind et al. 2000; Michaud et al. 2006; Benesh and Hafer 2012). Shortly, we took a photo of each parasite within its host and measured the area the parasite occupied using image J (Rasband 2008). From this we calculated the proportion of the non-infective parasites from day 7 among the total parasite area.

## Results

### Parasite size

Size could be measured for a total of 38 copepods with one parasite from day 0 and a varying number of parasites from day 7 (19 with 1 parasite from day 7, 16 with 2 parasites from day 7, 5 with 3 parasites from day 7). In copepods with one parasite from day 0 and one parasite from day 7, the parasite from day 7 made up 23 +/- 2%, significantly less than 50%, of the total area parasites occupied within these copepods (t=-11.7, df=18, p<0.0001, Fig. S3). In copepods harboring more than 1 non-infective parasite from day 7, the area these parasites occupied out of the total parasite area was not significantly different from 50% (2 parasites from day 7: 48 +/- 4 %, t=-1.3087, df=15, p=0.2103, 3 parasites from day 7: 49 +/- 8 %, t=-0.3561, df=4, p=0.7397, Fig. S3).

### Behavioral experiment

Neither the number of parasites a copepod was infected by on day 7 nor its interaction with day had any effect of how often it moved (Fig. S3). Additionally including the interaction between parasite number and time point in the recording (i.e. before vs. after a simulated predation attack) and the three way interaction including also the day significantly improved the model (Table S6). Post-hoc tests however, revealed no differences between pairwise comparisons between the activity of copepods with different numbers of parasites from day 7 (Table S7).

## References

Benesh, D. P., and N. Hafer. 2012. Growth and ontogeny of the tapeworm *Schistocephalus solidus* in its copepod first host affects performance in its stickleback second intermediate host. Parasit. Vectors 5.

Michaud, M., M. Milinski, G. A. Parker, and J. C. Chubb. 2006. Competitive growth strategies in intermediate hosts: Experimental tests of a parasite life-history model using the cestode, *Schistocephalus solidus*. Evol. Ecol. 20:39–57.

Rasband, W. S. 2008. ImageJ. U.S. National Institutes of Health, Bethesda, Maryland, USA.

Wedekind, C., M. Christen, L. Schärer, and N. Treichel. 2000. Relative helminth size in crustacean hosts: in vivo determination, and effects of host gender and within-host competition in a copepod infected by a cestode. Aquat. Ecol. 279–285.
