# Supplementary material for: When parasites disagree: Evidence for parasite-induced sabotage of host manipulation
Source: Evolution. 2015 Mar 10;69(3):611–20. doi: 10.1111/evo.12612 (PMC4409835; doi:10.1111/evo.12612)
Supplement: Supplementary file 7 — Table S1. Outcome of likelihood ratio tests. [file evo0069-0611-sd7.doc]

**Table S1: Outcome of likelihood ratio tests.** All comparisons were significant. The initial model used whether or not a copepod moved within a two second interval as response and the day after the first infection (DAY), the Period in the recording (PERIOD), i.e. after a simulated predation attack vs. after a recovery period and the interaction between DAY and TIME as fixed effects. We used the copepod identity as a random factor and included DAY and PERIOD. Subsequently, we added the treatment (TREAT) and all its interactions with DAY and PERIOD. Test statistics and MCMC-estimated p-values are for the comparison with the preceding model.

| Experiment 1 | | | |  | Experiment 2 | | | |
| --- | --- | --- | --- | --- | --- | --- | --- | --- |
|  |  |  |  |  |  |  |  |  |
| Factors | DF | Chisq | p |  | Factors | DF | Chisq | p |
| + TREAT | 15,5 | 29.381 | <0.0001 |  | + TREAT | 14,4 | 24.650 | <0.0001 |
| + DAY:TREAT | 20,5 | 15.459 | 0.0086 |  | + DAY:TREAT | 18,4 | 11.083 | 0.0257 |
| + PERIOD:TREAT | 25,5 | 138.240 | <0.0001 |  | + PERIOD:TREAT | 22,4 | 71.088 | <0.0001 |
| + PERIOD:DAY:TREAT | 30,5 | 119.914 | <0.0001 |  | + PERIOD:DAY:TREAT | 26,4 | 13.233 | 0.0102 |
|  |  |  |  |  |  |  |  |  |
| 63060 observations on 147 copepods | | | |  | 49980 observations on 121 copepods | | | |
