# Supplementary material for: When parasites disagree: Evidence for parasite-induced sabotage of host manipulation
Source: Evolution. 2015 Mar 10;69(3):611–20. doi: 10.1111/evo.12612 (PMC4409835; doi:10.1111/evo.12612)
Supplement: Supplementary file 8 — Table S2. Outcome of multiple comparisons between days for each treatment and period in the recording (i.e., after a simulated predation attack vs. after a recovery period). [file evo0069-0611-sd8.doc]

**Table S2: Outcome of multiple comparisons between days for each treatment and period in the recording (i.e. after a simulated predation attack vs. after a recovery period). Results from experiment 1. Significant p-values are highlighted in bold. C: uninfected control copepods, Sing_t0: copepods singly infected with one parasite on day 0, Sim_t0: copepods simultaneously infected with two parasites on day 0, Sing_t7: copepods singly infected with one parasite on day 7, Sim_t7: copepods simultaneously infected with two parasites on day 7, Seq: copepods sequentially infected with two parasites, one each on day 0 plus day 7.**

| After simulated predation attack | | | | | | | | | | | | |
| --- | --- | --- | --- | --- | --- | --- | --- | --- | --- | --- | --- | --- |
| Treatment | C | | Sing_t0 | | Sing_t7 | | Sim_t0 | | Sim_t7 | | Seq | |
|  | | | | | | | | | | | | |
| Comparison | Z | p | Z | p | Z | p | Z | p | Z | p | Z | p |
| day9-day11 | -0.48 | 1.000 | 4.53 | **<0.001** | -3.87 | **0.003** | 4.31 | **<0.001** | -4.42 | **<0.001** | 5.37 | **<0.001** |
| day11-day13 | -1.89 | 0.556 | 4.60 | **<0.001** | -2.87 | 0.078 | 5.79 | **<0.001** | -2.56 | 0.170 | 3.07 | **0.045** |
| day13-day15 | 1.89 | 0.554 | 0.43 | 1.000 | -2.18 | 0.365 | 1.78 | 0.632 | 2.82 | 0.089 | -0.49 | 1.000 |
| day15-day17 | -1.86 | 0.575 | 0.95 | 0.981 | 4.56 | **<0.001** | -1.42 | 0.848 | 3.82 | **0.003** | 3.40 | **0.015** |
| day17-day19 | 1.27 | 0.912 | -2.24 | 0.327 | 4.40 | **<0.001** | 0.65 | 0.998 | 9.60 | **<0.001** | -2.52 | 0.188 |
| day19-day21 | -0.14 | 1.000 | 3.88 | **0.003** | 1.94 | 0.521 | -2.78 | 0.098 | -1.08 | 0.960 | 0.08 | 1.000 |
| day21-day23 | -5.25 | **<0.001** | -0.21 | 1.000 | -1.79 | 0.627 | -2.55 | 0.174 | -0.22 | 1.000 | -2.82 | 0.091 |
|  | | | | | | | | | | | | |
| Observations | 8610 | | 5370 | | 5760 | | 2610 | | 5280 | | 3900 | |
| Copepods | 41 | | 25 | | 27 | | 11 | | 25 | | 18 | |
|  | | | | | | | | | | | | |
| After a recovery period | | | | | | | | | | | | |
| Treatment | C | | Sing_t0 | | Sing_t7 | | Sim_t0 | | Sim_t7 | | Seq | |
|  | | | | | | | | | | | | |
| Comparison | Z | p | Z | p | Z | p | Z | p | Z | p | Z | p |
| day9-day11 | -0.63 | 0.998 | 1.93 | 0.529 | -9.90 | **<0.001** | -0.80 | 0.993 | -8.74 | **<0.001** | 5.91 | **<0.001** |
| day11-day13 | 4.30 | **<0.001** | 3.57 | **0.009** | -1.22 | 0.927 | 6.17 | **<0.001** | 1.73 | 0.670 | -1.45 | 0.835 |
| day13-day15 | -0.87 | 0.989 | 1.25 | 0.918 | -0.96 | 0.980 | 0.41 | 1.000 | -1.80 | 0.617 | -2.51 | 0.192 |
| day15-day17 | -2.53 | 0.184 | -3.66 | **0.006** | 0.70 | 0.997 | 2.93 | 0.065 | 0.00 | 1.000 | 4.36 | **<0.001** |
| day17-day19 | -3.08 | **0.043** | -2.84 | 0.086 | 4.97 | **<0.001** | -7.49 | **<0.001** | 8.37 | **<0.001** | 0.81 | 0.993 |
| day19-day21 | 4.81 | **<0.001** | 3.40 | **0.015** | -4.03 | **0.002** | 3.05 | **0.047** | -1.46 | 0.831 | -3.31 | **0.020** |
| day21-day23 | -6.27 | **<0.001** | -0.71 | 0.997 | -4.21 | **0.001** | -3.38 | **0.016** | -0.33 | 1.000 | 2.34 | 0.271 |
|  | | | | | | | | | | | | |
| Observations | 8610 | | 5370 | | 5760 | | 2610 | | 5280 | | 3900 | |
| Copepods | 41 | | 25 | | 27 | | 11 | | 25 | | 18 | |
