# Supplementary material for: When parasites disagree: Evidence for parasite-induced sabotage of host manipulation
Source: Evolution. 2015 Mar 10;69(3):611–20. doi: 10.1111/evo.12612 (PMC4409835; doi:10.1111/evo.12612)
Supplement: Supplementary file 9 — Table S3. Outcome of multiple comparisons between treatments for each day and period in the recording (i.e., after a simulated predation attack vs. after a recovery period). [file evo0069-0611-sd9.doc]

**Table S3: Outcome of multiple comparisons between treatments for each day and period in the recording (i.e. after a simulated predation attack vs. after a recovery period). Results from experiment 1. Significant p-values are highlighted in bold. C: uninfected control copepods, Sing_t0: copepods singly infected with one parasite on day 0, Sim_t0: copepods simultaneously infected with two parasites on day 0, Sing_t7: copepods singly infected with one parasite on day 7, Sim_t7: copepods simultaneously infected with two parasites on day 7, Seq: copepods sequentially infected with two parasites, one each on day 0 plus day 7.**

| After simulated predation attack | | | | | | | | | | | | | | | | |
| --- | --- | --- | --- | --- | --- | --- | --- | --- | --- | --- | --- | --- | --- | --- | --- | --- |
| Day | 9 | | 11 | | 13 | | 15 | | 17 | | 19 | | 21 | | 23 | |
|  | | | | | | | | | | | | | | | | |
| Comparison | Z | p | Z | p | Z | p | Z | p | Z | p | Z | p | Z | p | Z | p |
| C-Sing_t0 | -3.29 | **0.013** | -1.38 | 0.733 | 0.43 | 0.998 | -0.37 | 0.999 | 0.70 | 0.982 | -0.27 | 1.000 | 1.02 | 0.908 | 2.18 | 0.243 |
| C-Sing_t7 | -1.70 | 0.526 | -3.15 | **0.020** | -3.13 | **0.021** | -5.08 | **<0.001** | -2.65 | 0.083 | -1.04 | 0.901 | -0.54 | 0.994 | -0.04 | 1.000 |
| C-Sim_t0 | -2.24 | 0.216 | 0.01 | 1.000 | 2.61 | 0.091 | 2.78 | *0.059* | 2.00 | 0.340 | 2.08 | 0.292 | 1.27 | 0.798 | 1.47 | 0.681 |
| C-Sim_t7 | -1.42 | 0.712 | -3.36 | **0.010** | -3.42 | **0.008** | -3.26 | **0.014** | -1.07 | 0.890 | 2.30 | 0.188 | 1.56 | 0.620 | 2.69 | 0.076 |
| C-Seq | -2.41 | 0.148 | -0.11 | 1.000 | 1.41 | 0.714 | 0.96 | 0.929 | 2.10 | 0.285 | 0.88 | 0.950 | 1.18 | 0.843 | 1.38 | 0.733 |
| Sing_t0-Sing_t7 | 1.51 | 0.654 | -1.54 | 0.633 | -3.13 | **0.021** | -4.13 | 0.001 | -3.03 | **0.028** | -0.67 | 0.984 | -1.45 | 0.690 | -1.99 | 0.346 |
| Sing_t0-Sim_t0 | 0.23 | 1.000 | 0.99 | 0.921 | 2.11 | 0.277 | 2.85 | 0.049 | 1.35 | 0.754 | 2.14 | 0.260 | 0.43 | 0.998 | -0.30 | 1.000 |
| Sing_t0-Sim_t7 | 1.67 | 0.547 | -1.77 | 0.475 | -3.38 | **0.009** | -2.55 | 0.108 | -1.61 | 0.588 | 2.31 | 0.185 | 0.55 | 0.994 | 0.52 | 0.995 |
| Sing_t0-Seq | 0.46 | 0.997 | 1.00 | 0.915 | 0.90 | 0.945 | 1.17 | 0.846 | 1.35 | 0.753 | 1.03 | 0.905 | 0.20 | 1.000 | -0.53 | 0.995 |
| Sing_t7-Sim_t0 | -0.94 | 0.933 | 2.21 | 0.226 | 4.63 | **<0.001** | 6.20 | **<0.001** | 3.85 | **0.002** | 2.72 | 0.070 | 1.62 | 0.580 | 1.39 | 0.731 |
| Sing_t7-Sim_t7 | 0.21 | 1.000 | -0.28 | 1.000 | -0.20 | 1.000 | 1.56 | 0.618 | 1.46 | 0.684 | 3.06 | **0.026** | 1.94 | 0.373 | 2.45 | 0.136 |
| Sing_t7-Seq | -0.89 | 0.948 | 2.40 | 0.153 | 3.87 | **0.001** | 5.03 | **<0.001** | 4.14 | **<0.001** | 1.65 | 0.556 | 1.58 | 0.605 | 1.29 | 0.785 |
| Sim_t0-Sim_t7 | 1.09 | 0.884 | -2.40 | 0.153 | -4.84 | **<0.001** | -4.92 | **<0.001** | -2.68 | 0.077 | -0.31 | 1.000 | 0.04 | 1.000 | 0.74 | 0.977 |
| Sim_t0-Seq | 0.16 | 1.000 | -0.09 | 1.000 | -1.30 | 0.779 | -1.75 | 0.495 | -0.13 | 1.000 | -1.13 | 0.865 | -0.25 | 1.000 | -0.18 | 1.000 |
| Sim_t7-Seq | -1.05 | 0.898 | 2.60 | 0.095 | 4.12 | **<0.001** | 3.56 | **0.005** | 2.84 | *0.050* | -1.02 | 0.908 | -0.33 | 0.999 | -0.99 | 0.918 |
|  | | | | | | | | | | | | | | | | |
| Observations | 4200 | | 4200 | | 4170 | | 4140 | | 3840 | | 3780 | | 3750 | | 3450 | |
| Copepods | 140 | | 140 | | 139 | | 138 | | 128 | | 126 | | 125 | | 115 | |
|  | | | | | | | | | | | | | | | | |
| After a recovery period | | | | | | | | | | | | | | | | |
| Day | 9 | | 11 | | 13 | | 15 | | 17 | | 19 | | 21 | | 23 | |
|  | | | | | | | | | | | | | | | | |
| Comparison | Z | p | Z | p | Z | p | Z | p | Z | p | Z | p | Z | p | Z | p |
| C-Sing_t0 | -3.05 | **0.027** | -1.89 | 0.401 | -2.35 | 0.172 | -2.16 | 0.250 | -2.34 | 0.177 | -2.27 | 0.200 | -2.52 | 0.115 | -0.90 | 0.947 |
| C-Sing_t7 | -0.86 | 0.955 | -3.22 | **0.016** | -4.93 | **<0.001** | -6.36 | **<0.001** | -4.68 | **<0.001** | -2.01 | 0.331 | -4.27 | **<0.001** | -3.90 | **0.001** |
| C-Sim_t0 | -2.05 | 0.310 | -1.91 | 0.387 | -0.79 | 0.968 | -0.78 | 0.970 | 0.80 | 0.966 | -1.15 | 0.857 | -1.07 | 0.890 | -1.30 | 0.784 |
| C-Sim_t7 | -0.88 | 0.951 | -3.22 | **0.016** | -3.96 | **0.001** | -5.11 | **<0.001** | -3.47 | **0.007** | -0.10 | 1.000 | -1.86 | 0.421 | -0.78 | 0.970 |
| C-Seq | -3.33 | **0.011** | -0.81 | 0.965 | -1.63 | 0.574 | -3.10 | **0.023** | -0.98 | 0.923 | -0.58 | 0.992 | -2.08 | 0.295 | 0.03 | 1.000 |
| Sing_t0-Sing_t7 | 2.05 | 0.308 | -1.15 | 0.855 | -2.28 | 0.196 | -3.65 | **0.003** | -2.15 | 0.259 | 0.32 | 1.000 | -1.56 | 0.620 | -2.73 | 0.068 |
| Sing_t0-Sim_t0 | 0.23 | 1.000 | -0.46 | 0.997 | 0.93 | 0.937 | 0.85 | 0.956 | 2.49 | 0.124 | 0.59 | 0.992 | 0.90 | 0.946 | -0.53 | 0.995 |
| Sing_t0-Sim_t7 | 1.93 | 0.374 | -1.22 | 0.821 | -1.38 | 0.733 | -2.61 | 0.092 | -1.01 | 0.911 | 1.99 | 0.339 | 0.47 | 0.997 | 0.07 | 1.000 |
| Sing_t0-Seq | -0.59 | 0.991 | 0.75 | 0.975 | 0.45 | 0.998 | -1.03 | 0.906 | 1.05 | 0.898 | 1.29 | 0.788 | 0.28 | 1.000 | 0.77 | 0.972 |
| Sing_t7-Sim_t0 | -1.36 | 0.748 | 0.44 | 0.998 | 2.77 | 0.061 | 3.81 | **0.002** | 4.24 | **<0.001** | 0.34 | 0.999 | 2.19 | 0.240 | 1.78 | 0.472 |
| Sing_t7-Sim_t7 | -0.05 | 1.000 | -0.10 | 1.000 | 0.94 | 0.935 | 0.99 | 0.918 | 1.15 | 0.859 | 1.74 | 0.500 | 1.95 | 0.369 | 2.72 | 0.070 |
| Sing_t7-Seq | -2.44 | 0.138 | 1.77 | 0.475 | 2.57 | 0.103 | 2.31 | 0.187 | 3.03 | **0.029** | 1.04 | 0.903 | 1.76 | 0.485 | 3.26 | **0.014** |
| Sim_t0-Sim_t7 | 1.29 | 0.786 | -0.51 | 0.996 | -2.04 | 0.312 | -2.98 | **0.034** | -3.33 | **0.011** | 1.01 | 0.911 | -0.47 | 0.997 | 0.58 | 0.992 |
| Sim_t0-Seq | -0.70 | 0.982 | 1.04 | 0.901 | -0.51 | 0.996 | -1.67 | 0.547 | -1.47 | 0.677 | 0.54 | 0.994 | -0.63 | 0.988 | 1.16 | 0.855 |
| Sim_t7-Seq | -2.33 | 0.176 | 1.83 | 0.442 | 1.73 | 0.502 | 1.38 | 0.737 | 1.99 | 0.343 | -0.46 | 0.997 | -0.18 | 1.000 | 0.68 | 0.984 |
|  | | | | | | | | | | | | | | | | |
| Observations | 4200 | | 4200 | | 4170 | | 4140 | | 3840 | | 3780 | | 3750 | | 3450 | |
| Copepods | 140 | | 140 | | 139 | | 138 | | 128 | | 126 | | 125 | | 115 | |
