# Supplementary material for: When parasites disagree: Evidence for parasite-induced sabotage of host manipulation
Source: Evolution. 2015 Mar 10;69(3):611–20. doi: 10.1111/evo.12612 (PMC4409835; doi:10.1111/evo.12612)
Supplement: Supplementary file 10 — Table S4. Outcome of multiple comparisons between days for each treatment and period in the recording (i.e., after a simulated predation attack vs. after a recovery period). [file evo0069-0611-sd10.doc]

**Table S4: Outcome of multiple comparisons between days for each treatment and period in the recording (i.e. after a simulated predation attack vs. after a recovery period).** Results from experiment 2. Significant p-values are highlighted in bold. C: uninfected control copepods, Sing_t0: copepods singly infected with one parasite on day 0, Sing_t7: copepods singly infected with one parasite on day 7, Seq: copepods sequentially infected with two parasites, one each on day 0 plus day 7, Seq2: copepods sequentially infected with three parasites, one on day 0 plus two on day 7.

| After simulated predation attack | | | | | | | | | | |
| --- | --- | --- | --- | --- | --- | --- | --- | --- | --- | --- |
| Treatment | C | | Sing_t0 | | Sing_t7 | | Seq | | Seq2 | |
|  | | | | | | | | | | |
| Comparison | Z | p | Z | p | Z | p | Z | p | Z | p |
| day9-day11 | -3.37 | **0.013** | 2.99 | **0.045** | -2.98 | **0.045** | -1.86 | 0.505 | -2.82 | 0.071 |
| day11-day13 | 1.55 | 0.715 | 3.01 | **0.043** | 0.93 | 0.968 | 7.27 | **<0.001** | 3.30 | **0.017** |
| day13-day15 | 1.12 | 0.921 | 2.46 | 0.174 | -1.35 | 0.829 | -1.42 | 0.792 | 1.07 | 0.937 |
| day15-day17 | 0.61 | 0.997 | -0.70 | 0.993 | 2.78 | 0.080 | 5.76 | **<0.001** | 6.32 | **<0.001** |
| day17-day19 | -0.90 | 0.972 | -1.97 | 0.435 | 4.28 | **<0.001** | -2.79 | 0.077 | 0.87 | 0.976 |
| day19-day21 | 0.55 | 0.998 | 1.52 | 0.736 | 1.28 | 0.863 | -3.87 | **0.002** | 0.80 | 0.985 |
|  | | | | | | | | | | |
| Observations | 4140 | | 5130 | | 4590 | | 5790 | | 5340 | |
| Copepods | 20 | | 25 | | 22 | | 28 | | 26 | |
|  | | | | | | | | | | |
| After a recovery period | | | | | | | | | | |
| Treatment | C | | Sing_t0 | | Sing_t7 | | Seq | | Seq2 | |
|  | | | | | | | | | | |
| Comparison | Z | p | Z | p | Z | p | Z | p | Z | p |
| day9-day11 | -2.81 | 0.074 | 1.51 | 0.739 | -5.43 | **<0.001** | -3.56 | **0.007** | -0.35 | 1.000 |
| day11-day13 | 3.62 | **0.006** | 0.00 | 1.000 | -6.22 | **<0.001** | 5.17 | **<0.001** | 2.32 | 0.233 |
| day13-day15 | -2.21 | 0.291 | 3.30 | **0.017** | 2.17 | 0.309 | -2.44 | 0.180 | 0.16 | 1.000 |
| day15-day17 | -0.36 | 1.000 | 0.35 | 1.000 | 1.54 | 0.722 | 2.50 | 0.161 | 0.56 | 0.998 |
| day17-day19 | -3.05 | **0.037** | -1.42 | 0.791 | 8.05 | **<0.001** | -2.82 | 0.071 | 3.72 | **0.004** |
| day19-day21 | 4.87 | **<0.001** | -1.75 | 0.584 | -2.16 | 0.319 | -2.42 | 0.191 | 0.07 | 1.000 |
|  | | | | | | | | | | |
| Observations | 4140 | | 5130 | | 4590 | | 5790 | | 5340 | |
| Copepods | 20 | | 25 | | 22 | | 28 | | 26 | |
