# Supplementary material for: When parasites disagree: Evidence for parasite-induced sabotage of host manipulation
Source: Evolution. 2015 Mar 10;69(3):611–20. doi: 10.1111/evo.12612 (PMC4409835; doi:10.1111/evo.12612)
Supplement: Supplementary file 11 — Table S5. Outcome of multiple comparisons between treatments for each day and period in the recording (i.e., after a simulated predation attack vs. after a recovery period). [file evo0069-0611-sd11.doc]

**Table S5: Outcome of multiple comparisons between treatments for each day and period in the recording (i.e. after a simulated predation attack vs. after a recovery period).** Results from experiment 2. Significant p-values are highlighted in bold. C: uninfected control copepods, Sing_t0: copepods singly infected with one parasite on day 0, Sing_t7: copepods singly infected with one parasite on day 7, Seq: copepods sequentially infected with two parasites, one each on day 0 plus day 7, Seq2: copepods sequentially infected with three parasites, one on day 0 plus two on day 7.

| After simulated predation attack | | | | | | | | | | | | | | |
| --- | --- | --- | --- | --- | --- | --- | --- | --- | --- | --- | --- | --- | --- | --- |
| Day | 9 | | 11 | | 13 | | 15 | | 17 | | 19 | | 21 | |
|  | | | | | | | | | | | | | | |
| Comparison | Z | p | Z | p | Z | p | Z | p | Z | p | Z | p | Z | p |
| C-Sing_t0 | -3.18 | **0.013** | -0.75 | 0.945 | -0.44 | 0.992 | 0.14 | 1.000 | -0.46 | 0.991 | -0.50 | 0.987 | -0.41 | 0.994 |
| C-Sing_t7 | -3.09 | **0.017** | -2.44 | 0.104 | -2.23 | 0.167 | -3.49 | **0.004** | -3.04 | **0.020** | -1.07 | 0.821 | -0.85 | 0.916 |
| C-Seq | -2.40 | 0.116 | -1.21 | 0.745 | 0.23 | 0.999 | -0.46 | 0.991 | 0.99 | 0.859 | 0.77 | 0.938 | -0.89 | 0.901 |
| C-Seq2 | -2.23 | 0.169 | -1.69 | 0.438 | -1.14 | 0.783 | -1.02 | 0.845 | 0.27 | 0.999 | 1.17 | 0.770 | 1.16 | 0.772 |
| Sing_t0-Sing_t7 | -0.01 | 1.000 | -1.80 | 0.372 | -1.92 | 0.306 | -3.81 | **0.001** | -2.75 | **0.047** | -0.61 | 0.973 | -0.45 | 0.992 |
| Sing_t0-Seq | 0.88 | 0.903 | -0.46 | 0.990 | 0.74 | 0.948 | -0.64 | 0.968 | 1.56 | 0.522 | 1.35 | 0.656 | -0.47 | 0.990 |
| Sing_t0-Seq2 | 1.05 | 0.832 | -0.99 | 0.858 | -0.75 | 0.946 | -1.23 | 0.733 | 0.78 | 0.937 | 1.76 | 0.399 | 1.64 | 0.473 |
| Sing_t7-Seq | 0.86 | 0.911 | 1.41 | 0.621 | 2.68 | *0.056* | 3.26 | **0.010** | 4.33 | **<0.001** | 1.94 | 0.296 | 0.01 | 1.000 |
| Sing_t7-Seq2 | 1.02 | 0.846 | 0.84 | 0.917 | 1.22 | 0.741 | 2.68 | *0.057* | 3.52 | **0.004** | 2.32 | 0.139 | 2.07 | 0.231 |
| Seq-Seq2 | 0.17 | 1.000 | -0.56 | 0.981 | -1.51 | 0.554 | -0.61 | 0.974 | -0.77 | 0.938 | 0.44 | 0.992 | 2.21 | 0.177 |
|  | | | | | | | | | | | | | | |
| Observations | 3600 | | 3600 | | 3600 | | 3570 | | 3540 | | 3540 | | 3540 | |
| Copepods | 120 | | 120 | | 120 | | 119 | | 118 | | 118 | | 118 | |
|  | | | | | | | | | | | | | | |
| After a recovery period | | | | | | | | | | | | | | |
| Day | 9 | | 11 | | 13 | | 15 | | 17 | | 19 | | 21 | |
|  | | | | | | | | | | | | | | |
| Comparison | Z | p | Z | p | Z | p | Z | p | Z | p | Z | p | Z | p |
| C-Sing_t0 | -4.42 | **<0.001** | -2.26 | 0.158 | -3.39 | **0.006** | -1.37 | 0.649 | -1.66 | 0.456 | -0.94 | 0.883 | -3.21 | **0.012** |
| C-Sing_t7 | -3.28 | **0.009** | -3.14 | **0.014** | -6.27 | **<0.001** | -4.56 | **<0.001** | -4.76 | **<0.001** | -0.77 | 0.940 | -3.36 | **0.007** |
| C-Seq | -3.65 | **0.002** | -2.50 | 0.089 | -2.50 | 0.090 | -2.47 | 0.097 | -2.00 | 0.267 | -1.32 | 0.676 | -3.88 | **0.001** |
| C-Seq2 | -4.54 | **<0.001** | -2.76 | **0.045** | -3.33 | **0.008** | -2.31 | 0.142 | -2.45 | 0.102 | -0.03 | 1.000 | -1.93 | 0.300 |
| Sing_t0-Sing_t7 | 1.09 | 0.813 | -1.01 | 0.852 | -3.24 | **0.011** | -3.40 | **0.006** | -3.35 | **0.007** | 0.16 | 1.000 | -0.20 | 1.000 |
| Sing_t0-Seq | 0.91 | 0.894 | -0.19 | 1.000 | 1.05 | 0.831 | -1.13 | 0.790 | -0.30 | 0.998 | -0.37 | 0.996 | -0.55 | 0.981 |
| Sing_t0-Seq2 | -0.09 | 1.000 | -0.54 | 0.984 | 0.10 | 1.000 | -0.96 | 0.871 | -0.83 | 0.922 | 0.96 | 0.871 | 1.42 | 0.617 |
| Sing_t7-Seq | -0.23 | 0.999 | 0.86 | 0.913 | 4.32 | **<0.001** | 2.40 | 0.115 | 3.18 | **0.013** | -0.53 | 0.984 | -0.34 | 0.997 |
| Sing_t7-Seq2 | -1.18 | 0.760 | 0.49 | 0.989 | 3.37 | **0.007** | 2.55 | 0.080 | 2.58 | 0.073 | 0.78 | 0.935 | 1.60 | 0.497 |
| Seq-Seq2 | -1.01 | 0.852 | -0.37 | 0.996 | -0.96 | 0.873 | 0.17 | 1.000 | -0.56 | 0.981 | 1.38 | 0.640 | 2.06 | 0.236 |
|  | | | | | | | | | | | | | | |
| Observations | 3600 | | 3600 | | 3600 | | 3570 | | 3540 | | 3540 | | 3540 | |
| Copepods | 120 | | 120 | | 120 | | 119 | | 118 | | 118 | | 118 | |
