# Supplementary material for: When parasites disagree: Evidence for parasite-induced sabotage of host manipulation
Source: Evolution. 2015 Mar 10;69(3):611–20. doi: 10.1111/evo.12612 (PMC4409835; doi:10.1111/evo.12612)
Supplement: Supplementary file 12 — Table S6. Outcome of likelihood ratio tests. [file evo0069-0611-sd12.doc]

**Table S6: Outcome of likelihood ratio tests.** Significant p-values are marked in bold. The initial model used whether or not a copepod moved within a two second interval as response and the day after the first infection (DAY), the Period in the recording (PERIOD), i.e. after a simulated predation attack vs. after a recovery period and the interaction between DAY and TIME as fixed effects. We used the copepod identity as a random factor and included DAY and PERIOD. Subsequently, we added the number of parasites a copepod was infected by on day 7 (NUMBER) and all its interactions with DAY and PERIOD. All copepods were infected by one parasite on day 0. Test statistics and MCMC-estimated p-values are for the comparison with the preceding model.

| Factors | DF | Chisq | p |
| --- | --- | --- | --- |
| + NUMBER | 11,1 | 0.303 | 0.5817 |
| + DAY:NUMBER | 12,3 | 0.675 | 0.4113 |
| + PERIOD: NUMBER | 13,1 | 19.355 | **<0.0001** |
| + PERIOD:DAY: NUMBER | 14,1 | 51.745 | **<0.0001** |
|  | | | |
| 22500 observations on 105 copepods | | | |
