# Supplementary material for: When parasites disagree: Evidence for parasite-induced sabotage of host manipulation
Source: Evolution. 2015 Mar 10;69(3):611–20. doi: 10.1111/evo.12612 (PMC4409835; doi:10.1111/evo.12612)
Supplement: Supplementary file 13 — Table S7. Outcome of multiple comparisons between treatments for each day and period in the recording (i.e., after a simulated predation attack (A) vs. after a recovery period (B)). [file evo0069-0611-sd13.doc]

**Table S7: Outcome of multiple comparisons between treatments for each day and period in the recording (i.e. after a simulated predation attack (A) vs. after a recovery period (B)). Significant p-values are highlighted in bold. The comparison gives the numbers of parasites copepods were infected by on day 7. Every copepod was infected additionally by one parasite on day 0. No significant differences occurred while there should have been a conflict between parasites that infected their copepod host on day 0 and on day 7 (day 11 to 15)**

| A: After simulated predation attack | | | | | | | | |
| --- | --- | --- | --- | --- | --- | --- | --- | --- |
| Day | 9 | | 11 | | 13 | | 15 | |
|  | | | | | | | | |
| Comparison | Z | p | Z | p | Z | p | Z | p |
| 0-1 | -2.62 | **0.041** | 0.83 | 0.834 | 0.87 | 0.815 | 0.79 | 0.856 |
| 0-2 | -1.14 | 0.659 | 2.20 | 0.117 | 1.33 | 0.538 | 1.34 | 0.533 |
| 0-3 | -0.98 | 0.754 | 1.75 | 0.289 | 2.23 | 0.111 | 0.80 | 0.849 |
| 1-2 | 1.52 | 0.419 | 1.60 | 0.371 | 0.54 | 0.947 | 0.65 | 0.914 |
| 1-3 | 0.79 | 0.856 | 1.29 | 0.562 | 1.71 | 0.309 | 0.30 | 0.991 |
| 2-3 | -0.20 | 0.997 | 0.25 | 0.994 | 1.31 | 0.548 | -0.15 | 0.999 |
|  | | | | | | | | |
| Observations | 3090 | | 2580 | | 2790 | | 2670 | |
| Copepods | 103 | | 86 | | 93 | | 89 | |
|  | | | | | | | | |
| B: After recovery period | | | | | | | | |
| Day | 9 | | 11 | | 13 | | 15 | |
|  | | | | | | | | |
| Comparison | Z | p | Z | p | Z | p | Z | p |
| 0-1 | -2.12 | 0.142 | -0.96 | 0.769 | -1.14 | 0.656 | -0.39 | 0.979 |
| 0-2 | -0.67 | 0.908 | 0.46 | 0.967 | -0.03 | 1.000 | 0.09 | 1.000 |
| 0-3 | -0.41 | 0.975 | 0.61 | 0.927 | -1.73 | 0.297 | -0.48 | 0.962 |
| 1-2 | 1.50 | 0.432 | 1.48 | 0.440 | 1.15 | 0.648 | 0.48 | 0.962 |
| 1-3 | 1.04 | 0.718 | 1.26 | 0.578 | -1.04 | 0.721 | -0.24 | 0.995 |
| 2-3 | 0.05 | 1.000 | 0.30 | 0.990 | -1.74 | 0.296 | -0.54 | 0.946 |
|  | | | | | | | | |
| Observations | 3090 | | 2580 | | 2790 | | 2670 | |
| Copepods | 103 | | 86 | | 93 | | 89 | |
